# Supplementary material for: Development of a computer-aided design software for the quantitative evaluation of aesthetic damage
Source: PLoS One. 2019 Dec 18;14(12):e0226322. doi: 10.1371/journal.pone.0226322 (PMC6919621; doi:10.1371/journal.pone.0226322)
Supplement: S1 Code site — 1. (ZIP) [file pone.0226322.s001.zip › [EESC_jr][Projeto]Relatorio_07_21/reports/templates_zip/medialoot-lumino-admin-bootstrap-template.zip!/Lumino Admin Bootstrap Template/lumino/icons.html]

Lumino - Icons


Toggle navigation

LuminoAdmin

- User 
  - Profile
  - Settings
  - Logout

- Dashboard
- Widgets
- Charts
- Tables
- Forms
- Alerts & Panels
- Icons
- Dropdown
  - Sub Item 1
  - Sub Item 2
  - Sub Item 3
- Login Page

2. Icons

# All Icons

**Stroked Icon Set** *(full set available via Glyphs.co)*

```
<svg class="glyph stroked app window with content"><use xlink:href="#stroked-app-window-with-content"/></svg>
```

```
<svg class="glyph stroked app-window"><use xlink:href="#stroked-app-window"></use></svg>
```

```
<svg class="glyph stroked bag"><use xlink:href="#stroked-bag"></use></svg>
```

```
<svg class="glyph stroked basket "><use xlink:href="#stroked-basket"/></svg>
```

```
<svg class="glyph stroked calendar blank"><use xlink:href="#stroked-calendar-blank"/></svg>
```

```
<svg class="glyph stroked calendar"><use xlink:href="#stroked-calendar"/></svg>
```

```
<svg class="glyph stroked camera "><use xlink:href="#stroked-camera"/></svg>
```

```
<svg class="glyph stroked camcorder"><use xlink:href="#stroked-camcorder"/></svg>
```

```
<svg class="glyph stroked cancel"><use xlink:href="#stroked-cancel"/></svg>
```

```
<svg class="glyph stroked checkmark"><use xlink:href="#stroked-checkmark"/></svg>
```

```
<svg class="glyph stroked clipboard with paper"><use xlink:href="#stroked-clipboard-with-paper"/></svg>
```

```
<svg class="glyph stroked clock"><use xlink:href="#stroked-clock"/></svg>
```

```
<svg class="glyph stroked dashboard dial"><use xlink:href="#stroked-dashboard-dial"/></svg>
```

```
<svg class="glyph stroked desktop"><use xlink:href="#stroked-desktop"/></svg>
```

```
<svg class="glyph stroked blank document"><use xlink:href="#stroked-blank-document"/></svg>
```

```
<svg class="glyph stroked download"><use xlink:href="#stroked-download"/></svg>
```

```
<svg class="glyph stroked eye"><use xlink:href="#stroked-eye"/></svg>
```

```
<svg class="glyph stroked open folder"><use xlink:href="#stroked-open-folder"/></svg>
```

```
<svg class="glyph stroked folder"><use xlink:href="#stroked-folder"/></svg>
```

```
<svg class="glyph stroked gear"><use xlink:href="#stroked-gear"/></svg>
```

```
<svg class="glyph stroked home"><use xlink:href="#stroked-home"/></svg>
```

```
<svg class="glyph stroked heart"><use xlink:href="#stroked-heart"/></svg>
```

```
<svg class="glyph stroked key "><use xlink:href="#stroked-key"/></svg>
```

```
<svg class="glyph stroked chain"><use xlink:href="#stroked-chain"/></svg>
```

```
<svg class="glyph stroked location pin"><use xlink:href="#stroked-location-pin"/></svg>
```

```
<svg class="glyph stroked lock"><use xlink:href="#stroked-lock"/></svg>
```

```
<svg class="glyph stroked map"><use xlink:href="#stroked-map"/></svg>
```

```
<svg class="glyph stroked empty message"><use xlink:href="#stroked-empty-message"/></svg>
```

```
<svg class="glyph stroked two messages"><use xlink:href="#stroked-two-messages"/></svg>
```

```
<svg class="glyph stroked mobile device"><use xlink:href="#stroked-mobile-device"/></svg>
```

```
<svg class="glyph stroked music"><use xlink:href="#stroked-music"/></svg>
```

```
<svg class="glyph stroked notepad "><use xlink:href="#stroked-notepad"/></svg>
```

```
<svg class="glyph stroked paperclip"><use xlink:href="#stroked-paperclip"/></svg>
```

```
<svg class="glyph stroked landscape"><use xlink:href="#stroked-landscape"/></svg>
```

```
<svg class="glyph stroked plus sign"><use xlink:href="#stroked-plus-sign"/></svg>
```

```
<svg class="glyph stroked printer"><use xlink:href="#stroked-printer"/></svg>
```

```
<svg class="glyph stroked sound on"><use xlink:href="#stroked-sound-on"/></svg>
```

```
<svg class="glyph stroked star"><use xlink:href="#stroked-star"/></svg>
```

```
<svg class="glyph stroked table"><use xlink:href="#stroked-table"/></svg>
```

```
<svg class="glyph stroked tablet"><use xlink:href="#stroked-tablet-1"/></svg>
```

```
<svg class="glyph stroked tag"><use xlink:href="#stroked-tag"/></svg>
```

```
<svg class="glyph stroked trash"><use xlink:href="#stroked-trash"/></svg>
```

```
<svg class="glyph stroked monitor"><use xlink:href="#stroked-monitor"/></svg>
```

```
<svg class="glyph stroked upload"><use xlink:href="#stroked-upload"/></svg>
```

```
<svg class="glyph stroked male user "><use xlink:href="#stroked-male-user"/></svg>
```

```
<svg class="glyph stroked film"><use xlink:href="#stroked-film"/></svg>
```

```
<svg class="glyph stroked female user"><use xlink:href="#stroked-female-user"/></svg>
```

```
<svg class="glyph stroked video"><use xlink:href="#stroked-video"/></svg>
```

```
<svg class="glyph stroked arrow down"><use xlink:href="#stroked-arrow-down"/></svg>
```

```
<svg class="glyph stroked arrow left"><use xlink:href="#stroked-arrow-left"/></svg>
```

```
<svg class="glyph stroked arrow right"><use xlink:href="#stroked-arrow-right"/></svg>
```

```
<svg class="glyph stroked arrow up"><use xlink:href="#stroked-arrow-up"/></svg>
```

```
<svg class="glyph stroked chevron down"><use xlink:href="#stroked-chevron-down"/></svg>
```

```
<svg class="glyph stroked chevron left"><use xlink:href="#stroked-chevron-left"/></svg>
```

```
<svg class="glyph stroked chevron right"><use xlink:href="#stroked-chevron-right"/></svg>
```

```
<svg class="glyph stroked chevron up"><use xlink:href="#stroked-chevron-up"/></svg>
```

```
<svg class="glyph stroked desktop computer and mobile"><use xlink:href="#stroked-desktop-computer-and-mobile"/></svg>
```

```
<svg class="glyph stroked flag"><use xlink:href="#stroked-flag"/></svg>
```

```
<svg class="glyph stroked external hard drive"><use xlink:href="#stroked-external-hard-drive"/></svg>
```

```
<svg class="glyph stroked internal hard drive"><use xlink:href="#stroked-internal-hard-drive"/></svg>
```

```
<svg class="glyph stroked hourglass"><use xlink:href="#stroked-hourglass"/></svg>
```

```
<svg class="glyph stroked laptop computer and mobile"><use xlink:href="#stroked-laptop-computer-and-mobile"/></svg>
```

```
<svg class="glyph stroked open letter"><use xlink:href="#stroked-open-letter"/></svg>
```

```
<svg class="glyph stroked email"><use xlink:href="#stroked-email"/></svg>
```

```
<svg class="glyph stroked brush"><use xlink:href="#stroked-brush"/></svg>
```

```
<svg class="glyph stroked pencil"><use xlink:href="#stroked-pencil"/></svg>
```

```
<svg class="glyph stroked toiler paper"><use xlink:href="#stroked-toiler-paper"/></svg>
```

```
<svg class="glyph stroked usb flash drive"><use xlink:href="#stroked-usb-flash-drive"/></svg>
```

```
<svg class="glyph stroked pen tip"><use xlink:href="#stroked-pen-tip"/></svg>
```

```
<svg class="glyph stroked wireless router"><use xlink:href="#stroked-wireless-router"/></svg>
```

```
<svg class="glyph stroked round coffee mug"><use xlink:href="#stroked-round-coffee-mug"/></svg>
```

```
<svg class="glyph stroked paper coffee cup"><use xlink:href="#stroked-paper-coffee-cup"/></svg>
```

```
<svg class="glyph stroked bacon burger"><use xlink:href="#stroked-bacon-burger"/></svg>
```

```
<svg class="glyph stroked line-graph"><use xlink:href="#stroked-line-graph"></use></svg>
```
